# Using a smartphone on the move: do visual constraints explain why we slow walking speed?

**DOI:** 10.1007/s00221-021-06267-6

**Published:** 2021-11-18

**Authors:** Alejandro Rubio Barañano, Muhammad Faisal, Brendan T. Barrett, John G. Buckley

**Affiliations:** 1 School of Optometry and Vision Science, University of Bradford, Bradford, UK; 2 Faculty of Health Studies, University of Bradford, Bradford, UK; 3 Department of Biomedical and Electronics Engineering, University of Bradford, Bradford, UK

**Keywords:** Smart-phone, Reading performance, Head motion, Vision, Walking

## Abstract

Viewing one’s smartphone whilst walking commonly leads to a slowing of walking. Slowing walking speed may occur because of the visual constraints related to reading the hand-held phone whilst in motion. We determine how walking-induced phone motion affects the ability to read on-screen information. Phone-reading performance (PRP) was assessed whilst participants walked on a treadmill at various speeds (Slow, Customary, Fast). The fastest speed was repeated, wearing an elbow brace (Braced) or with the phone mounted stationary (Fixed). An audible cue (‘text-alert’) indicated participants had 2 s to lift/view the phone and read aloud a series of digits. PRP was the number of digits read correctly. Each condition was repeated 5 times. 3D-motion analyses determined phone motion relative to the head, from which the variability in acceleration in viewing distance, and in the point of gaze in space in the up-down and right-left directions were assessed. A main effect of condition indicated PRP decreased with walking speed; particularly so for the Braced and Fixed conditions (*p *= 0.022). Walking condition also affected the phone’s relative motion (*p *< 0.001); post-hoc analysis indicated that acceleration variability for the Fast, Fixed and Braced conditions were increased compared to that for Slow and Customary speed walking (*p *≤ 0.05). There was an inverse association between phone acceleration variability and PRP (*p *= 0.02). These findings may explain why walking speed slows when viewing a hand-held phone: at slower speeds, head motion is smoother/more regular, enabling the motion of the phone to be coupled with head motion, thus making fewer demands on the oculomotor system. Good coupling ensures that the retinal image is stable enough to allow legibility of the information presented on the screen.

**Supplementary Information:**

The online version contains supplementary material available at 10.1007/s00221-021-06267-6.

## Introduction

The worldwide rate of smartphone usage is estimated to be in the billions and continues to increase annually, from an estimated 3.6 billion users in 2016 to 6.4 billion users in 2021 (O’Dea 2021). It is very common to see people using their smartphone whilst they move about the environment. There is considerable previous research investigating the effects on walking of concurrently using a smartphone while reading, texting or talking, (Schabrun et al. 2014; Kao et al. 2015; Alsaleh et al. 2018; Crowley et al. 2016, 2019). The findings from these studies show that key walking parameters change as a consequence of concurrent smartphone usage, including reducing walking speed with a shortened and wider step. Using a phone whilst walking represents a cognitive dual task, the execution of which has been shown to indirectly affect walking speed (Crowley et al. 2016; Krasovsky et al. 2017) and this could explain why walking speed slows when using a smartphone. It is also possible however, that because viewing a phone blocks part of the visual scene and means the individual is not looking where they would normally look as they walk, users slow their walking speed because of an increased risk of tripping over, or colliding with, obstacles in the environment. There is evidence that using a smartphone whilst walking causes an increased tripping risk (Schabrun et al. 2014; Kao et al. 2015; Alsaleh et al. 2018; Crowley et al. 2016, 2019), which is presumably why injuries have been found to be more common among pedestrians using a phone compared to pedestrians not using a phone (Nasar and Troyer 2013).

While the effects of smartphone use on walking have been studied, there is currently a paucity of research investigating how the act of walking might affect the ability to use a smartphone, for example, the ability to read the text presented on the screen. The current study is concerned with understanding how walking-induced phone motion affects the ability to accurately read information presented on the phone screen.

Previous studies of vision under dynamic conditions have typically determined the smallest letter/symbol size that can be resolved, when either the target being viewed is in motion whilst the participant is static (Ludvigh and Miller 1958; Miller 1958), or when the participant’s head is rotating whilst viewing a static target (Roberts et al. 2006; Vital et al. 2010). Findings from these studies indicate that, although a certain amount of target and/or head motion can be tolerated without a detrimental effect on visual acuity (Westheimer and McKee 1975), in general visual acuity decays as a function of increasing speed of target or of head rotation (Ludvigh and Miller 1958; Miller 1958; Roberts et al. 2006; Vital et al. 2010; Westheimer and McKee 1975). A disadvantage of these approaches in assessing dynamic vision is that they do not reflect the dynamic conditions where both the individual and the target being viewed are in motion. Such dynamic situations represent a complicated visual challenge; an example of which is when viewing/reading information on a hand-held smartphone while the user is concurrently walking. In this situation both the head and phone are in motion.

When walking, the motion of the head can be described as rhythmic, with the extent and frequency related to the walking speed (Verbecque et al. 2018), and with higher walking speeds inducing increased motion with a more variable head-acceleration pattern (Latt et al. 2008). Walking induces not only head motion, but also motion of the freely swinging arms, meaning that motion of a hand-held phone will be different to that of the head and hence the eyes. The motion of the phone relative to the eyes will create varying demands of the gaze-control systems to stabilise the retinal image of the information presented on the smartphone screen. Holding a phone while walking will also mean that the distance (in depth direction) between the phone and the eyes will vary (Schabrun et al. 2014). This variation in depth may prompt the need for greater and lesser amounts of accommodation, as the phone-to-eyes distance reduces and increases, respectively. Depending on the size of the detail being viewed, not being able to adjust to changes in the accommodative demand quickly enough as the phone moves (in depth direction) could cause the information on the screen to appear out of focus.

Having a stable, or a relatively stable, retinal image is also important for achieving good visual performance. When retinal image motion (horizontal or vertical) is greater than 2.5 degrees/second, the ability to discriminate fine detail decays (Westheimer and McKee 1975). When viewing a stationary object while the head is moving, the vestibular-ocular reflex (VOR) triggers compensatory eye movement in the opposite direction to stabilise the retinal image (Leigh and Zee 1999). However, whilst walking and viewing a smartphone, stabilising the retinal image will likely require additional eye movements to those induced by the VOR alone (Das et al. 1999; King and Shanidze 2011). The need to stabilise the retinal image of the screen can be reduced by coupling the motion of the phone to the motion of the head. Motion-coupling arises when the movements of two body segments are co-ordinated within an overall movement pattern (Donker et al. 2001; Garg et al. 2014; Hamill et al. 2020). Hence if the motion of the hand-held phone is coupled with the walking-induced motion of the head, this would minimise the extent to which the VOR, saccades and pursuit eye movements are needed. If motion of the hand-held phone was perfectly coupled with head motion, then only saccadic eye movements would be necessary for reading. However, perfect coupling is unlikely to ever occur, so reading a phone on the move will always require compensatory eye movements. A drive to couple the motion of the phone to motion of the eyes, to minimize the compensatory eye movements that would otherwise be required, could explain why pedestrians slow their speed when viewing the screen on their smartphone.

Previous research investigating dynamic visual acuity (DVA) whilst reading a smartphone when walking has reported that reducing the phone-to-eyes distance, or increasing the font size of the text/numbers helps to compensate for the reduction in visual acuity that is induced by walking (either overground or on a treadmill), but reducing walking speed by itself did not improve DVA (Conradi and Alexander 2014). In a study undertaken in the Nokia Research Centre, phone-text legibility was shown to be affected by speed of walking, with improved legibility at slower speeds (Mustonen et al. 2004). However, the latter study provided no insights as to why phone-text legibility was better at the slower speeds.

The aim of the present study was to determine how increases in the phone-to-eyes relative motion during walking affects the ability to read information displayed on a smartphone. The approach we take here is to measure smartphone reading performance when the motion of the phone relative to the eyes is modulated. This was achieved in two ways: (a) by gradually increasing the walking speed which induced increased head and hand (phone) motion, and (b) walking whilst wearing an elbow brace or walking with the phone mounted on the treadmill (static), which restricted or eliminated, respectively, the ability to couple the movement of the hand-held phone with the motion of the eyes.

We hypothesised that, because head motion would be relatively low and regular when walking at slow and customary speeds, participants would be able to achieve good coupling between the motion of the hand-held phone and motion of the eyes, i.e., phone-to-eyes relative motion would be relatively smooth and regular, so that reading information from the screen would be achieved with similar ability as when standing still. However, with faster walking, particularly when wearing an elbow brace or having a stationary phone, it would be difficult, or impossible, to achieve good coupling between the motion of the phone and motion of the eyes, and thus phone-to-eyes relative motion would become erratic, resulting in substantial decrements in reading performance. With erratic phone-to-eyes relative motion, the decrements in reading performance would result from an inability to make the necessary eye movements and/or adjustments in accommodation to achieve a sufficiently stable and focussed retinal image for the task to be effectively executed.

## Materials and methods

### Participants

Twenty healthy individuals (10 males, 10 females; 25.1 ± 4.3 years; height, 1.7 ± 0.1 m; mass, 71.6 ± 22.1 kg) took part in the study. Participants were excluded if they were older than 35 years, had consumed alcohol in the last 24 h, had any injury or musculoskeletal disorder or were taking medicines that might affect gait, balance or posture. They were also excluded if their visual acuity of either eye (assessed with the Snellen chart, MacLure 1980, Clement Clarke International Ltd.—London, UK) with the refractive correction habitually worn outdoors (either single-vison spectacles or contact lenses) was worse than 6/9 (0.18 logMAR) at distance (6 m) or near (35 cm), or if stereoacuity was worse than 100″ of arc. Stereoacuity was assessed at 40 cm using the ‘Random dot’ test (Stereo Optical Co. Inc., Chicago, IL, EEUU). All participants owned a smartphone and indicated they regularly used it whilst walking. Motor eye dominance was determined using the Dolman method (hole-in-the-card test, Fink 1938). Sixteen participants (80%) were found to be right eye dominant and four were left eye dominant, and all were right-handed.

Ethical approval was obtained from the University of Bradford’s Committee for Ethics in Research and the tenets of the Declaration of Helsinki were followed. All participants gave informed and written consent prior to data collection.

### Experimental protocol

Participants were asked to attend the laboratory wearing shoes and clothes appropriate and comfortable for walking.

Each participant was first familiarised with walking on the laboratory treadmill (M-mill, ForceLink—Culemborg, The Netherlands) for a period of 2–5 min. Each participant’s customary walking speed was then determined using the approach described by Jordan et al. (2007). When the treadmill was started, the belt speed was incrementally increased until the participant indicated they were walking at their ‘normal’ walking speed. The treadmill speed was then increased further to a noticeably fast speed (from the participant’s perspective), after which it was incrementally decreased until, once again, the participant indicated the speed was ‘normal’ for them. The average of the two recorded values was taken as the participant’s customary speed (Cust).

### Phone-reading task

**Fig. 1:**
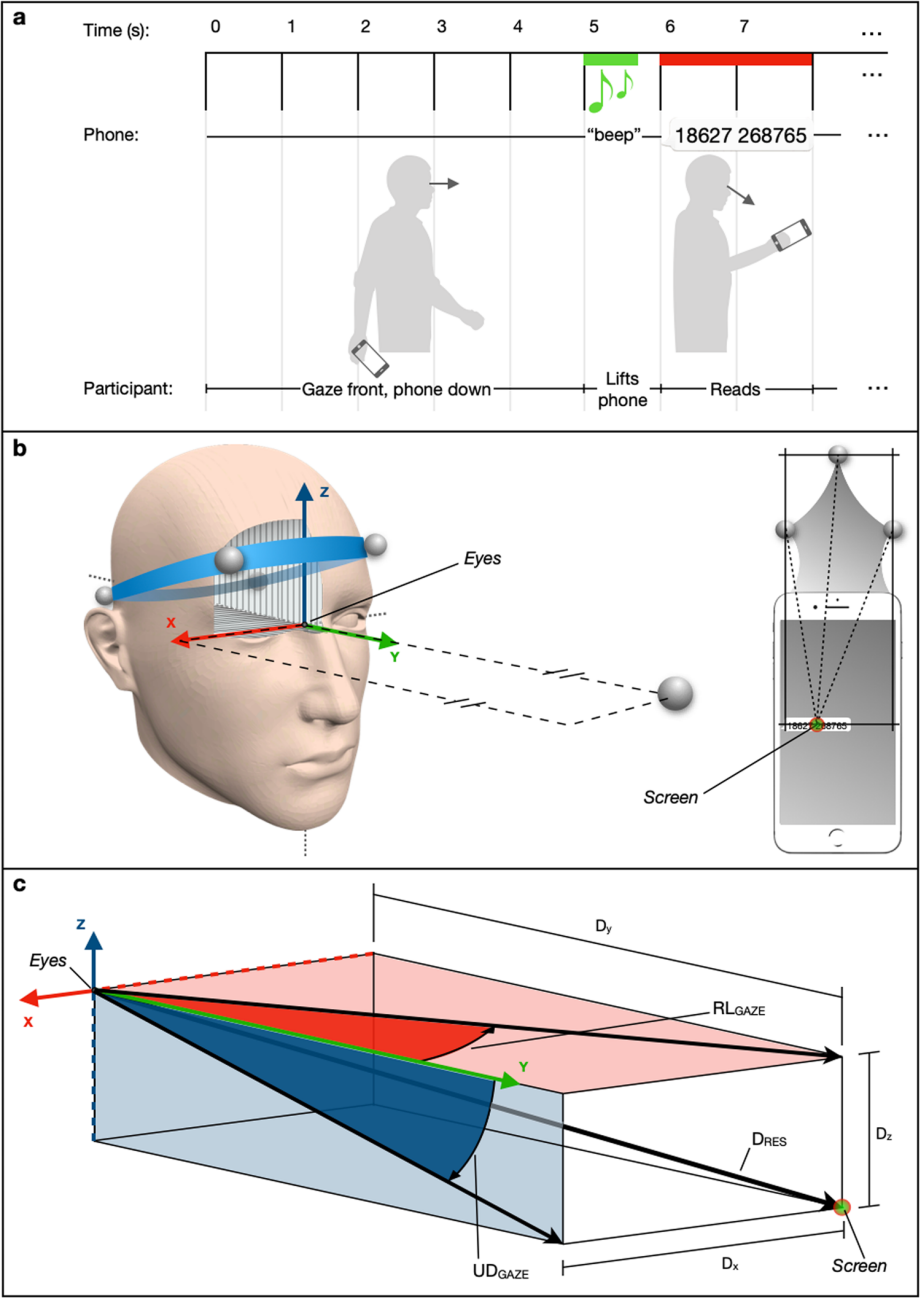
**a** The phone-reading task. Participants look to the front, holding the phone with arms by the side in relaxed position, as in normal walking. After hearing the “beep” from the phone, participants raise their arm, look at the screen, and begin reading aloud the sequence of numbers. The presentation disappears after 2 s, and participants look again to the front with arms relaxed (free to swing). 5 s later, the next reading trial commences until a total of 5 trials is completed in each block. **b** Static calibration. Defining the position of the *Eyes* and reference frame relative to the tracked “head” segment (headband), undertaken whilst participants stood still and looked to a marker placed at 1.5 m in front of them. The marker was horizontally and vertically aligned with the midpoint of their eyes. On the right-hand side, defining *Screen* position in relation to the markers attached to the “phone”, Note *Screen* offset, from middle of screen, represents location of where text is displayed. **c** Kinematic outcome measures: D_RES_ is distance of the *Screen* from the *Eyes,* i.e., resultant displacement (D_x_, D_y_, D_z_). UD_GAZE_ and RL_GAZE_ indicate gaze angles in the “*yz*” (up-down) and “*xy*” (left–right) planes, respectively, i.e., gaze angles relative to the head-segment’s reference frame (indicated by red [*x*] and blue [*z*] planes). The example in the figure shows the *Screen* displaced downwards and leftwards from the neutral (static calibration) alignment, resulting in negative UD_GAZE_ and RL_GAZE_ angles

An automated presentation simulating how one of the most widely used chat-apps displays text on a phone screen (i.e., a WhatsApp^®^ chat) was created for use on a smartphone (iPhone 7—Apple Inc., Cupertino, CA, EEUU). The presentation was an array of 11 single-digit numbers displayed in random order, in the format of a UK-style phone number (5 digits—space–6 digits, e.g., 79,564 348,967) and using a font type and size frequently used in iOS (Helvetica Neue, 38 pt., requiring a visual acuity of 6/24 or better based on the digits’ height and a 30–35 cm viewing distance). Figure 1a shows how each trial was run.

When participants were on the treadmill, they were asked (except for Fixed phone condition) to hold the phone in their preferred hand (right hand for all 20 participants) with the arms relaxed and free to swing, as in normal walking. A colour image (with no fine details) of a green pasture under a blue sky (Microsoft’s Windows XP desktop image) printed onto A4 size paper was placed at 5–6 m distance in front of the treadmill at a height of 1.6 m. For all conditions (including Fixed), participants were asked to look forward to the picture until a ‘beep’ (lasting 750 ms) from the hand-held (or stationary) phone was heard, simulating the situation in which a ‘new message’ had been received. On hearing the beep, participants were asked to lift the phone and to view the screen (Fig. 1a). All participants were able to hear the phone ‘beep’ in spite of the treadmill noise.

The visual stimulus appeared one second after the ‘beep’ started and remained on the screen for 2 s. Presentation timings were checked using an oscilloscope during pilot work and were found to be accurate within ± 20 ms of the intended 2 s duration. Screen brightness was set at 100%. The phone was turned to ‘do not disturb’ mode to avoid possible distractions (other notifications, calls, etc.).

Participants were asked to read aloud and in sequence (from left to right) the numbers that appeared on the screen. They were instructed to speak loudly enough so that they could be heard by the researcher. They were asked to name each digit separately, for example saying ‘one-two’ rather than ‘twelve’, or ‘zero-zero’ rather than ‘double-zero’. No more than 3 repeated digits were displayed in any one ‘message’. Once the stimulus disappeared from the screen, participants were instructed to return their arms to their sides (as in normal walking), and to direct their gaze frontwards once again. After 5 s, the next reading trial commenced until a block of 5 trials had been completed.

Participants undertook a block of 5 practice trials whilst walking at their customary walking speed. The phone-reading task was then completed for the following six conditions in a pseudo-random order: standing still (Standing), walking at customary speed (Cust), walking at 80% of customary speed (Slow), walking at 130% of customary speed (Fast), walking at the fast speed whilst wearing an elbow brace on the right arm (ROM Elbow Brace; Praxis Medical Ltd., Brierfield, Lancashire, UK) with the angle at the elbow set at 105 degrees (Braced), and walking at the fast speed with the phone mounted in a static position (i.e., not hand-held) in front of the participant at a height just above the top edge of the treadmill’s front rail, at their preferred distance (typically 40 cm away from the participant) (Fixed).

Phone-reading performance (PRP) was recorded using a sheet showing the correct set of numbers for every condition and trial. As the participant called out the numbers, the researcher recorded each correctly named digit. The total number of digits (out of 11) correctly read was the PRP for that trial. If a digit was missed from the sequence or misread but the remaining numbers were read correctly, this was recorded as a single-digit mistake; such errors were very infrequent (< 1% of trials). Decrements in PRP were invariably due to failing to read the numbers in the latter part of the sequence (due to running out of time) rather than reading out an incorrect number.

### Kinematic (motion) analysis

Infrared, reflective markers were attached to the head, via a headband. The markers were placed approximately over the left and right temples, and over the left and right posterolateral aspects of the head (Fig. 1b). Three infrared markers were also attached to a plastic card fixed to the phone (Fig. 1b). A six-camera motion analysis system (Vicon Bonita, Oxford Metrics PLC, Oxford, UK) was used to track and record marker motion in 3D space (at 100 Hz) as participants first completed a static calibration trial followed by each of the six conditions described above. The static calibration trial was recorded with each participant standing still whilst looking at a marker placed 1.5 m in front of them. This marker was horizontally and vertically aligned with the midpoint of their eyes when the head and eyes were directed straight ahead (neutral head and gaze position).

The motion data files collected from each participant, including static calibration, were post-processed using the Nexus 2.8 software in the following manner. Markers were reconstructed and labelled, and any gaps in their trajectories were filled. Data were then filtered using a low-pass digital filter (Butterworth) with a cut-off frequency of 6 Hz. The four head markers were joined to form a ‘head’ segment, and the three markers attached to the phone formed a ‘phone’ segment.

#### Determination of phone motion relative to the head

##### Setting the head-segment reference frame at the eyes’ midpoint

From the data collected for the static calibration, we set the origin of the head-segment’s reference frame (axes) at the midpoint of the eyes by undertaking the following steps within the ProCalc software. From the midpoint of the two front head markers, a vertical line was projected 4 cm down to the nasal bridge: pilot work had indicated that 4 ± 0.5 cm was typical for the eye-plane location relative to the headband. A horizontal offset of 1 cm towards the back of the head was then applied to match the approximated depth in the skull where the eyes are located. A virtual marker (*Eyes*) was then created at this location. The head’s reference frame (origin of axes) was embedded at the *Eyes* (Fig. 1b) with its axes rotated to match the ‘neutral gaze angles’ for the static calibration position (see Online Resource 1). Once embedded for this neutral position, the reference frame would obviously move when the head moved, i.e., in the dynamic trials.

A virtual marker (*Screen*) representing the position of the midpoint of the stimuli on the screen was also created. The location of this point was determined by measuring its distance from the ‘phone’ segment markers (Fig. 1b).

##### Assessing gaze without tracking eye movements

To measure the point of gaze in space, we assumed that when participants were reading the digits on the phone, their gaze was directed at the midpoint of where the digits were displayed. Thus, by tracking the movement of the phone in the head-segment’s reference frame (i.e., relative to its position when gaze was ‘neutral’), the angular changes in assumed gaze that occurred during the phone-reading period were measured. We assumed that during the period when the participant was reading the numbers, any relative movement of the *Screen* in the head’s *x–z* plane (Fig. 1c) would mean that the point of gaze in space must have been shifted leftwards or rightwards from neutral. Similarly, any relative movement of the *Screen* in the head’s *y–z* plane (Fig. 1c) must have meant point of gaze shifted upwards or downwards from neutral. In other words, since participants must direct their point of gaze at the phone screen to read the text presented on it, any up-down or right-left motion of the phone relative to the head during this period was assumed to cause a redirection of the point of gaze in space (i.e., changes in the assumed gaze angle in right-left and up-down directions). Measuring assumed gaze angle in this way can only be done for periods when it is known that the object being tracked (which in the current study was the phone) is being viewed. We discuss the limitations of this approach towards the end of the discussion.

##### Determination of phone-reading period

**Fig. 2:**
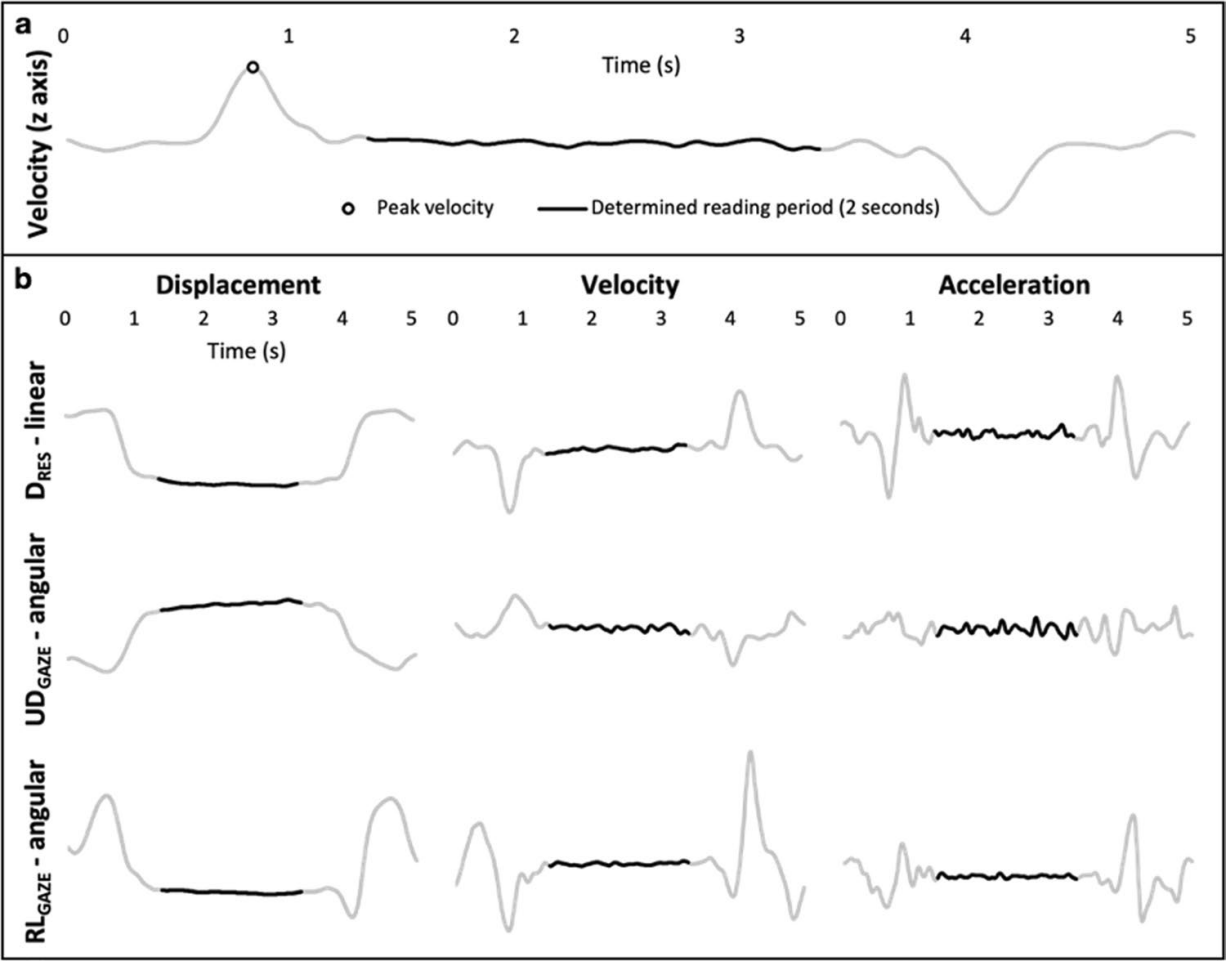
**a** Determination of phone-reading period: the beginning of each of the reading periods of 2 s was determined by finding the phone’s peak velocity (relative to the head) in the *z* axis (vertical direction) and adding 50 ms to the time at which peak velocity occurred. **b** Exemplar data from one participant showing the phone’s relative motion during a reading trial at customary speed. The black portion of line in each figure represents the 2 s ‘stable’ period, i.e., phone-reading period. The outcome variables (D_RES__Acc-SD, UD_GAZE__Acc-SD and RL_GAZE__Acc-SD) were determined for this ‘stable’ period only. The left-hand panels show the linear and angular displacement of the phone relative to the eyes; D_RES_, indicates the resultant, relative linear displacement, i.e., viewing distance, and UD_GAZE_ and RL_GAZE_, indicate the relative angular displacement of the phone in the Up-Down and Right-Left directions respectively. The middle and right-hand panels show the associated velocities and accelerations, i.e., the first and second derivatives of the displacements shown in the left-hand panels

The phone-reading period was assumed to have started following the instant the phone had been raised to read the digits displayed on the screen. This was determined using the following procedure. The first derivative of the phone’s linear displacement (relative to the head) in the vertical direction was determined (phone relative vertical velocity, *z* axis) (Fig. 2a). The local maximum in the relative vertical velocity (peak/zenith) was then located and an offset of 50 ms was added. The resulting time-point was considered as ‘onset of reading period’. The 50 ms offset was included following pilot work which indicated the phone’s relative vertical velocity had reduced to near-zero after 50 ms or less following the local maxima across all conditions. Outcome measures that described how the phone was moving relative to the head were determined for only the period from ‘onset of reading phase’ up to 2 s later, i.e., the period when the digits to be read were displayed on the screen.

For the Fixed phone condition, participants did not hold the phone so the method described above to determine the onset of each reading phase could not be used. Instead, participants were asked to touch their right shoulder with their right hand when they heard the phone ‘beep’. Touching their shoulder simulated the ‘phone-lifting’ that occurred in the other conditions. For these trials, an extra marker was placed on their right hand. By analysing the motion of this marker, a similar procedure to that described above was applied to find the onset of reading for this condition.

##### Kinematic outcome measures determined for phone-reading period

Viewing distance (*D*_*RES*_). Determined as the resultant linear displacement of the *Screen* relative to the *Eyes* position (Fig. 1c) using the formula:

$$D_{RES} = \sqrt {D_{x}^{ 2} + D_{y}^{ 2} + D_{z}^{ 2} } ,$$

where *D*_*x*_, *D*_*y*_, and *D*_*z*_ indicate the relative phone displacement in the *X*, *Y*, and *Z* directions.

From the *D*_*RES*_ data and using a 5-point moving window, we then determined the velocity (D_RES__Vel) and acceleration (D_RES__Acc) of changes in viewing distance.

Right-left and up-down assumed gaze angles (RL_GAZE,_ UD_GAZE_).

As described above (see *‘Assessing gaze without eye-tracking’*), any up-down or right-left motion of the phone relative to the head was assumed to cause a redirection of the point of gaze in space in right-left and up-down directions, respectively (Fig. 1c). Therefore, the assumed gaze angles were determined in degrees using the following formulae:

$$UD_{GAZE} = \tan^{ - 1} \frac{{D_{Z} }}{{D_{y} }}\cdot\left( {180/\pi } \right)$$

$$RL_{GAZE} = \tan^{ - 1} \frac{{D_{x} }}{{D_{y} }}\cdot\left( {180/\pi } \right),$$

where *D*_*x*_, *D*_*y*_, and *D*_*z*_ indicate the relative phone displacement in the *X*, *Y*, and *Z* directions, respectively.

From the *UD*_*GAZE*_ and *RL*_*GAZE*_ and using a 5-point moving window, we then determined the angular velocity (UD_GAZE__Vel, RL_GAZE__Vel) and acceleration (UD_GAZE__Acc, RL_GAZE__Acc) of the assumed gaze angle changes.

Perturbations in acceleration have been widely used to analyse how the act of walking affects dynamic stability (Menz et al. 2003; Kavanagh et al. 2004, 2005; Latt et al. 2008). Thus, in the current study our statistical analysis focuses on determining if, or how, the phone’s acceleration relative to the eyes differed across the different conditions. To this end, we determined the variability in the phone’s relative acceleration (relative to the head). Variability was determined as the standard deviation (SD) of the fluctuations in acceleration of the resultant phone-to-head distance (D_RES__Acc-SD), and in the Up-Down (UD_GAZE__Acc-SD) and Right-Left (RL_GAZE__Acc-SD) assumed gaze angles for each phone-reading period.

For descriptive purposes we also determined the group mean (± SD) phone-to-eyes distance, and the mean Up-Down (UD) and Right-Left (RL) gaze angles.

### Statistical analysis

To determine if outcome measures differed across conditions, data were analysed using repeated measures ANOVA, with condition and repetition as repeated measures factors. We used JASP (University of Amsterdam, Amsterdam, The Netherlands) for statistical analysis. The same software was used to undertake Holm-corrected post-hoc analyses. As a null hypothesis procedure, p values below 0.05 were considered as statistically significant. Sphericity tests were performed and when necessary, a Greenhouse–Geisser correction was applied to correct the degrees of freedom. Thus, all the p values shown have been corrected where the assumption of sphericity was violated.

For any of the outcome measures that were found to differ significantly across conditions, we determined how the changes in that outcome measure were associated with PRP. As PRP is a count response variable, this was determined using generalized linear mixed-effects modelling with family Poisson distribution (Martin et al. 2019; Brown 2021). We developed and used separate models for each of the three acceleration measures (D_RES__Acc-SD, UD_GAZE__Acc-SD, RL_GAZE__Acc-SD). Each acceleration measure was included as a standardised independent variable with participant included as a random effect. We report intercept, slope and *p* values for each model. Data were analysed using an R package ‘lme4’ (R Core Team 2020).

Across the participant group PRP was seen, in general, to improve from trial one to trial four but was consistently poorer in the 5th trial compared with the previous 4 repetitions (i.e., on average there was at least one more reading mistake in trial 5 compared to all other trials). As participants were completing the reading task on the 5th trial, the word ‘Finished!’ appeared. Although the digits to be read had disappeared when this word appeared on the screen, the ability to complete the reading task on the 5th trial was believed to be impaired by the presentation of this text after the disappearance of the digits. This unanticipated finding suggested a systematic error that likely made participants lose their focus on the task. Therefore, data from the 5th trial of every condition for all participants were excluded from the analyses.

## Results

The group average positioning of the phone relative to the eyes during the phone-reading period, and the associated assumed gaze angles for the different testing conditions are shown in Table 1. Values in the first column (D_RES_) show the group mean distance (± SD) of the phone relative to the eyes. Values in the second and third columns show the group average mean (± SD) assumed gaze angles (UD_GAZE_ and RL_GAZE_) in the Up-Down and Right-Left directions, respectively. There was a main effect of condition found for average viewing distances (D_RES_; *p *< 0.001). Post-hoc follow-up indicated that D_RES_ for the Braced and Fixed conditions were significantly greater than all other conditions (*p *< 0.001), and that D_RES_ for the Fixed condition was significantly greater than for the Braced condition (*p *< 0.001). There was also a main effect of condition for the average assumed gaze angle in the right-left direction (RL_GAZE_; *p *= 0.009), and post-hoc analysis indicated that RL_GAZE_ for the Braced condition was significantly greater than all other conditions accept Fixed. There were no significant main effects for condition in the average up-down gaze angle (*p *= 0.557).

**Table 1:** Group mean (± SD) viewing distance (D_RES_) and assumed gaze angles (UD_Gaze_ and RL_Gaze_) across the six conditions

Mean ± SD	D_RES_ (cm)^a^	UD_GAZE_ (deg)	RL_GAZE_ (deg)^a^
Standing	33.1 ± 4.9	− 10.7 ± 8.9	0.2 ± 12.7
Slow	33.1 ± 4.8	− 10.1 ± 7.9	− 0.3 ± 13.5
Cust	33.3 ± 5.3	− 10.3 ± 8.6	0.9 ± 13.2
Fast	33.3 ± 5	− 10.4 ± 7.3	0.4 ± 13.7
Braced	43.8 ± 5.2^b^	− 10.9 ± 10.1	3.6 ± 13.1^c^
Fixed	50.4 ± 9.2^b^	− 12.2 ± 8.5	1.2 ± 14.2

Walking speeds (mean ± SD) (m/s): slow = 0.92 ± 0.16; customary = 1.14 ± 0.20; fast = 1.49 ± 0.26

^a^Main effect of condition. Post-hoc analyses: ^b^different to all other conditions (*p *< 0.001)

^c^Different to all other conditions except *Fixed* (*p *< 0.05)

Figure 2b presents exemplar data from one participant showing the phone’s relative motion during a trial at customary walking speed. We focussed our statistical analysis on investigating how the phone’s acceleration relative to the eyes differed across the different walking conditions. This is because we knew that changes in the phone’s relative acceleration would precede any changes occurring in its relative velocity or displacement. Figure 2b highlights the merits of focussing on investigating changes in the phone’s relative acceleration, i.e., it highlights that the fluctuations in the acceleration trajectory are more evident than those occurring in either the velocity or displacement trajectories.

### Phone-reading performance (PRP)

**Fig. 3:**
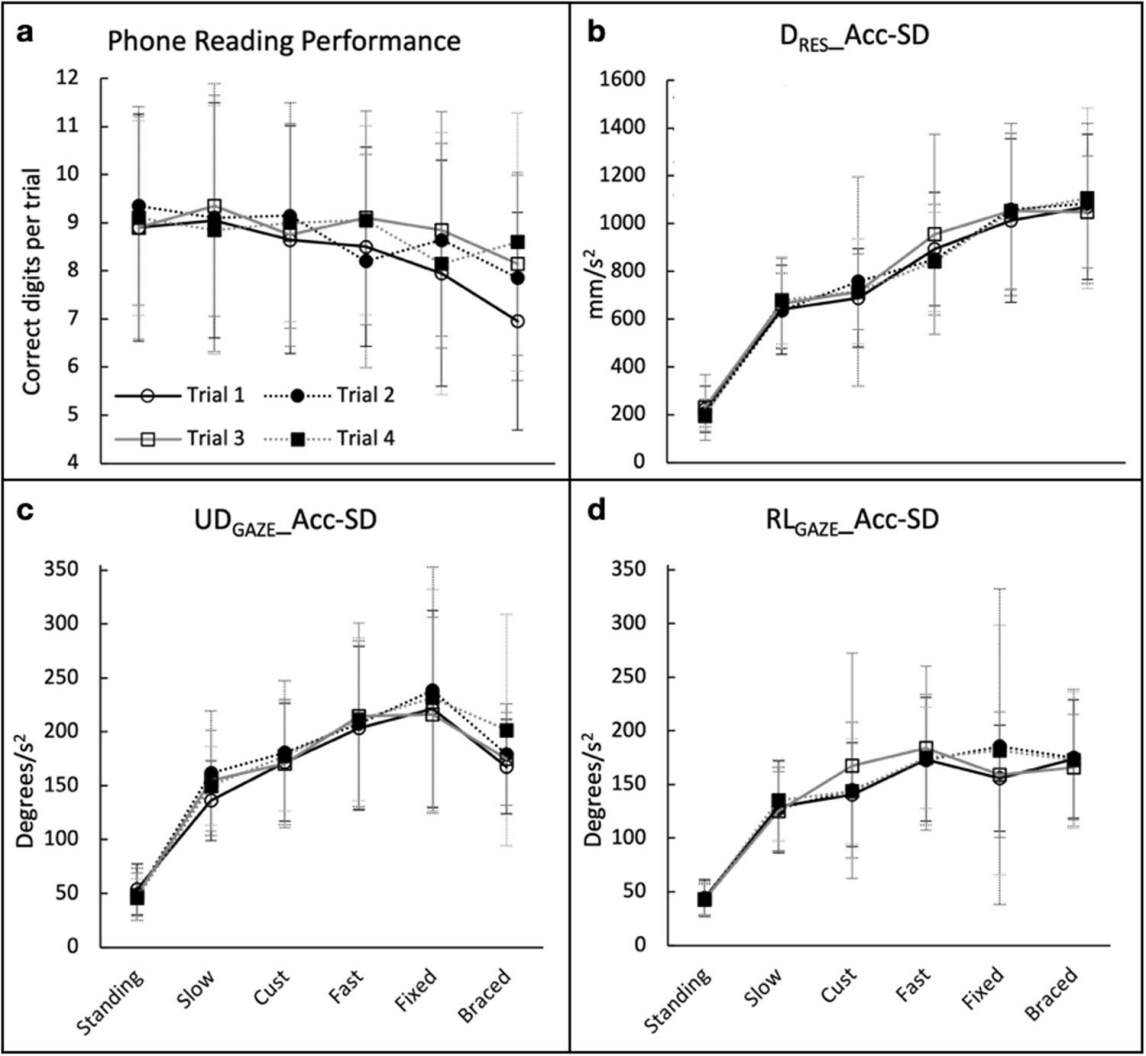
**a** Group mean PRP for each trial and condition. Error bars represent the group SD for each condition and trial. **b**–**d** Group average variability in the phone’s relative acceleration. **b** D_RES__Acc-SD; **c** UD_GAZE__Acc-SD; **d** RL_GAZE__Acc-SD across testing conditions and trial repetitions. Error bars represent the group SD for each condition and trial

Group average PRP across each of the six conditions is shown in Fig. 3a. The figure highlights how PRP decreased with increased walking speed, and it decreased further for the Fixed condition and further still for the Braced condition. There was a significant main effect for condition (*p *= 0.022) and trial repetition (*p *= 0.015), but there was no interaction between terms (*p *= 0.318). The condition main effect indicated that PRP tended to become poorer at the fastest speed, particularly for the Fixed and Braced conditions, and post-hoc analysis indicated PRP in the Braced condition was significantly poorer compared to the Standing (*p *= 0.016) and the Slow (*p *= 0.013) conditions. None of the differences between the other conditions reached statistical significance (all *p *> 0.6). Post-hoc analysis indicated the average PRP achieved in the first trial was significantly poorer compared to the third (*p *= 0.021), suggesting a training effect in the task. However, PRP did not differ between any of the other trial repetitions.

### Effects of phone-to-head relative acceleration

Group average trial acceleration variability in the resultant phone-to-eyes distance (D_RES__Acc-SD), and in the Up-Down (UD_GAZE__Acc-SD) and Right-Left (RL_GAZE__Acc-SD) assumed gaze angles across the different conditions, are shown in Fig. 3b–d. Similar trends across the different testing conditions were found for group average velocity variability and group average displacement variability in the resultant phone-to-eyes distance (D_RES__Acc-SD) and in the Up-Down (UD_GAZE__Acc-SD) and Right-Left (RL_GAZE__Acc-SD) assumed gaze angles (see Online Resource 2).

There were main effects of condition for all outcome variables (D_RES__Acc-SD; UD_GAZE__Acc-SD; RL_GAZE__Acc-SD, *p *< 0.001), but there was no effect of repetition (*p *> 0.149) and no interactions between terms (*p *> 0.365). The condition main effect indicated D_RES__Acc-SD increased with walking speed and increased further in the Fixed condition, and further still in the Braced condition (Fig. 3b). UD_GAZE__Acc-SD and RL_GAZE__Acc-SD also increased across the conditions from Standing to Fixed, but was seen to reduce slightly for the Braced compared to the Fixed condition (Fig. 3b). Post-hoc analysis indicated that the variability in acceleration outcomes (D_RES__Acc-SD, UD_GAZE__Acc-SD, RL_GAZE__Acc-SD) for the Standing condition were significantly reduced compared to all the other conditions (*p *< 0.001) and that D_RES__Acc-SD for the Fast, Fixed and Braced conditions were significantly increased compared to Slow, and Cust conditions (*p *< 0.029). Also, UD_GAZE__Acc-SD for the Fast and Fixed conditions was significantly increased compared to Slow, and Cust conditions (*p *≤ 0.05). In addition, RL_GAZE__Acc-SD for the Fast, Fixed and Braced conditions was significantly increased compared to Slow condition (*p *≤ 0.007).

### Association between Phone Reading Performance and kinematic outcomes

D_RES__Acc-SD, UD_GAZE__Acc-SD, RL_GAZE__Acc-SD were, in general, found to increase across conditions (from Standing to Braced, Fig. 3b–d), whilst PRP was found to decrease across the conditions (Fig. 3a). This suggested there was an association between PRP and the kinematics of the phone relative to the head. The generalized linear mixed-effects modelling we undertook determined the degree of association between PRP and each of the variability in acceleration outcome measures.

This analysis indicated a significant inverse (negative) association between D_RES__Acc-SD and PRP (slope, − 0.03845; intercept; 2.14317; *p *= 0.0204). However, the association between UD_GAZE__Acc-SD and PRP was not statistically significant (*p *= 0.283) though the association between RL_GAZE__Acc-SD and PRP did approach statistical significance (slope,  − 0.03693; intercept, 2.14349; *p *= 0.0549).

## Discussion

The aim of the present study was to determine how walking-induced motion of a hand-held phone in relation to the motion of the eyes, affects the ability to read text (numbers) displayed on the phone. Findings highlight that when walking on a flat and level treadmill at customary and slow speeds, motion of the phone relative to the eyes occurred in a smooth and regular manner (i.e., the phone’s acceleration relative to the eyes occurred with low variability), and as a result PRP was as good as it was when standing still. However, at faster walking speeds, and particularly when the elbow of the arm holding the phone was braced or when the phone was mounted stationary on the treadmill, motion of the phone relative to the eyes became irregular. This led to an increase in the variability of the phone’s relative acceleration (Fig. 3b–d) and, as a consequence, PRP became significantly poorer (Fig. 3a).

We speculated that a drive to couple the motion of the phone to motion of the eyes (see ‘‘Introduction’’), to minimize the eye movements that would otherwise be required to stabilise the retinal image, may explain why pedestrians slow their speed when reading their phones. The finding that at customary walking speed, relative phone motion was comparatively smooth and regular and PRP was as good as it is standing still, would suggest there is no need to slow customary walking speed to read information presented on a phone. Why then do we see people slowing down as they view their phones? In the present study, participants read their phone whilst walking on a constant-speed and level treadmill. Walking on a treadmill is different to walking overground in a few key respects. Firstly, on a treadmill, obviously there is no requirement to make locomotive adjustments to control heading direction and/or for the avoidance of approaching obstacles. Secondly, walking on a powered treadmill changes the biomechanics of walking (Song and Hidler 2008; Malatesta et al. 2017). Both these aspects are likely to make walking more metronomic and thus make motion of the phone relative to the eyes more predictable and thus more regular than that which occurs when walking at the same speed overground. Furthermore, previous research has shown that self-selected ‘normal’ walking speed is slightly slower on a treadmill than in overground walking (∆ ≈ 0.2 m/s) (Malatesta et al. 2017), and this speed difference may mean the present study underestimated the impact of customary speed walking on the ability to read information from the screen. We believe that this difference may explain why in the present study there was little effect on PRP at the customary walking speed. The limitations of using a treadmill are discussed further below, along with discussion of all findings from the study. This is one of only a few studies (Mustonen et al. 2004; Barnard et al. 2005; Schildbach and Rukzio 2010; Ng et al. 2014) to investigate how the act of walking impacts on the ability to process visual information presented on a phone screen. Understanding how walking affects the ability to use a phone is important, because smartphone usage is estimated to be over 6 billion worldwide (O’Dea 2021) and it is extremely common to see people using their phone whilst moving around their environment. It is worth highlighting again that most of the errors in PRP were due to participants not being able to read out all 11 digits because of running out of time, and < 1% of the errors were due to reading the digits incorrectly. Obviously, it would be expected that PRP scores would improve if the allotted reading period had been longer than 2 s. However, having a longer reading period would have partly negated the effects of the different walking conditions. By undertaking pilot testing, we surmised that the combination of the chosen letter size and chosen reading period would allow us to examine the visual constraints involved in this scenario, i.e., would allow us to determine how walking-induced phone motion affects the ability to read on-screen information.

Our findings indicate that PRP was poorer when there was increasing variability in the acceleration in viewing distance and in the assumed gaze angle in the right-left direction. This suggests that the visual system could not make the necessary eye movements and/or changes in accommodation quickly enough within the allotted 2 s response time period to stabilize and/or focus the retinal image of the irregularly moving phone. We believe these findings could explain why pedestrians slow their walking when viewing their phones, i.e., they decrease walking speed because the head motion induced at slower speeds is smoother and more regular, making it easier to couple the motion of the hand-held phone to motion of the head. In turn, this helps to ensure the retinal image of the phone screen is stable enough and/or clear (in focus) enough to be legible. Related to the above, it is worth noting that the association between D_RES__Acc-SD and PRP was significant and there was a trend (*p *= 0.0549) between RL_GAZE__Acc-SD and PRP but the association between UD_GAZE__Acc-SD and PRP was non-significant (*p *= 0.283). This highlights that PRP was affected by perturbations in D_RES_ and RL_GAZE_ but was unaffected by perturbations in UD_GAZE_. It is possible that the orientation of the stimuli explains the association trend found between RL_GAZE__Acc-SD and PRP: as the digits on the phone screen (the stimuli) were presented horizontally, any perturbations in the right-left motion of the phone are likely to disrupt reading accuracy. Whereas, because of the way the eyes move from left to right when reading, perturbations in phone motion in the up-down plane would be expected to have a much reduced effect on reading performance. If the stimuli had been presented vertically, perturbations in phone motion in the up-down plane may have had a greater effect on PRP. However, future research is needed to confirm this. What explains the association found between D_RES__Acc-SD and PRP, i.e., why did reading performance diminish with increases in D_RES__Acc-SD? Previous research has shown that, under binocular conditions, both the accommodative (Heron et al. 2001) and the vergence (Chirre et al. 2015) systems require a minimum response time to re-fixate and re-focus a stimulus that is rapidly moving (changing size) in the depth plane. For example, when shifting gaze from targets placed at 0.42 m. to ones at 0.75 m. or vice versa, accommodation requires on average 400 ms response time to achieve a focussed image after the change in depth (Heron et al. 2001). Furthermore, for changes in viewing distance from far (e.g. 2.75 m) to near (e.g. 0.3 m), the vergence system needs around 800 ms response time to re-fixate the eyes (converge) at the new (closer) depth plane (Chirre et al. 2015). Thus a likely explanation for our findings is that it was related to having perturbations in phone motion in the depth direction that were faster than the accommodative and/or vergence systems could cope with. However, future work is needed to confirm or refute this.

PRP was poorest for the conditions that had the greatest variability in the phone’s relative acceleration, namely the Braced and Fixed conditions. What factors might explain these findings? One possibility relates to the fact that the average viewing distance for these conditions differed considerably by comparison with all the other conditions (Table 1). The fact that the viewing distance was greater for the Braced and Fixed conditions means that the angular size of the digits to be read was smaller for these conditions compared to all other conditions where the viewing distance was just over 33 cm (Table 1). In the current study, perhaps the angular subtense at the larger viewing distance was too small to allow the number digits to be accurately discriminated? We don’t believe this was the case, because even with the increased viewing distance for these conditions (Fixed, Braced), the digits would have been large enough to remain clearly visible to our participants who had normal, or corrected-to-normal, vision. The largest viewing distance occurred for the Fixed condition, which was on average 0.505 ± 0.092 m. Even over the group range in this distance, visual acuity (VA) would have been sufficient to discriminate the digits (i.e., at 0.4 m distance, VA is 0.54 logMAR; at 0.6 m distance, VA is 0.36 logMAR). Hence visual acuity limitations cannot explain the greater reduction in PRP for the Braced or Fixed conditions (Fig. 3a). Similarly, differences in accommodation demand between the Braced and Fixed conditions relative to the other conditions cannot explain the poorer PRP in these conditions. This is because the accommodative demand is less when the viewing distance is greater, and because the variation in accommodation demand was no greater for the Braced and Fixed conditions compared to the other conditions. In any case, the temporal responsiveness of the human accommodation system does not allow the accommodation to vary in real time as the distance between the phone and the user increases and decreases as part of the rhythmic walking pattern (Dubost et al. 2006). Thus, our results suggest that the poorer PRP in the Braced and Fixed conditions compared to other conditions was not due to the factors of accommodation or visual acuity but instead as a result of the increased variability in the phone’s relative acceleration in the forward-back, up-down and right-left planes (Fig. 3). This is likely to have exerted particular challenges on the eye movement control systems whose responsibilities are to coordinate the movements of the eyes to keep the image of the moving stimulus on or near the fovea (Leigh and Zee 1999).

Our findings indicate that PRP in the Slow and Cust conditions was similar to that for the Standing condition even though the variability in the phone’s relative acceleration was much higher in the Slow and Cust conditions compared to the Standing condition. This suggests that the visual system is resistant to some movement of the target being viewed. However, once this threshold in the phone’s relative acceleration is exceeded, PRP is compromised, presumably because the retinal image becomes unstable. This threshold is unlikely to have a fixed/definitive value and will likely vary depending on the characteristics of the visual task. For example, if the stimulus (digit) size is decreased, a reduction in PRP from that achieved under static conditions (i.e., standing) might become evident even at slow walking speeds. Moreover, similar to the outcomes from the studies by Ludvigh and Miller (1958), Demer and Amjadi (1993) and Verbecque et al. (2018), the results of the present study indicate large inter-individual variations in PRP as walking speed increased. For example, Participant 5 was able to maintain the same PRP for the *Fast* condition as that achieved for the Slow (mean PRP for both conditions was 10.5 digits), while Participant 6’s PRP for the *Fast* condition (mean 7.75 digits) was noticeably poorer than what was achieved for the Slow condition (mean 10.5 digits) (Online Resource 2). This highlights that some individuals demonstrate greater resistance to increases in phone relative acceleration, while others are more susceptible to acceleration increases. Future work should investigate whether certain population groups, e.g., older adults, those with vestibular disorders, and those with increased incidence of falling are more susceptible to acceleration increases, and whether such susceptibility predisposes them to having poorer visual control during walking and hence puts them at an increased risk of tripping or falling.

Previous research has shown that working memory may be affected during periods of acute exercise (Marchant et al. 2020). Thus, an alternative interpretation for the inter-individual variations (described above) relates to the differing impact that walking may have had on an individual’s working memory. However, this seems unlikely because all participants appeared to cope easily with the exercise intensity, even at the ‘fast’ walking speed.

A key factor in any dynamic visual task is the input of the vestibulo-ocular reflex (VOR). This mechanism detects head motion and compensates via inducing eye movements to keep the retinal image as stable as possible (Grossman et al. 1989; Leigh and Zee 1999). Healthy humans are able to stabilise their heads relatively well in terms of flexion and extension (i.e. sagittal plane motion; often referred to as pitch) during locomotion (Pozzo et al. 1990), and a later study by the same group showed that such head stabilisation mechanisms are impaired in patients with vestibular deficits (Pozzo et al. 1991). Hence an area for future work could be to determine if, and to what extent, phone-reading performance whilst walking is poorer in patients with vestibular deficits. The VOR is known to have different responses for its horizontal and vertical components (Dietrich and Wuehr 2019). Thus any future work investigating dynamic phone-reading performance in patients with vestibular deficits might consider comparing phone-reading performance when the text to be read is presented vertically to when it is presented horizontally. It might be that reading direction has a different effect for patients with vestibular deficits than it does for individuals with a properly functioning vestibular system.

### Assessing changes in gaze angles without a gaze tracking device

In the present study, we presented a new approach for assessing where the gaze is directed that did not require the use of a gaze tracking device. Typically, identifying where the eyes are looking (i.e., where gaze is directed) is evaluated using an eye-tracking device. These devices are head-mounted which permits analysing the gaze angle relative to the head whilst the participant is in motion. The approach used in the present study was to measure the *assumed* gaze angle. By tracking movements of the phone screen relative to the head’s reference axis with the origin located at the midpoint of the eyes, we assumed that motion of the screen relative to the head would mean there was a corresponding change in gaze angle. As highlighted above, measuring assumed gaze angle in this way can only be done for periods when it is known that the object being tracked (which in the current study was the phone) is actually being viewed. In the present study we knew participants had to be looking at the phone screen during the period of the test because they were calling out the numbers presented on it. Determining the changes in assumed gaze angle in the up-down and right-left directions during the two second period when the phone was being viewed indicated the gaze requirements of the phone-reading task, i.e., it indicated where the eyes were assumed to be directed during this 2 s period. However, this is not necessarily where the gaze was actually directed to at any instant in time because changes in the actual gaze direction may have lagged behind, or moved ahead, of the instantaneous/changing position of the phone screen. We are planning to undertake work to determine how closely changes in assumed gaze angles match changes in the actual angle (direction) of gaze. If the angle of assumed gaze matches closely the actual angle of gaze, the approach we have used here will offer an advantage over using an eye-tracking system because eye-trackers (typically consisting of some type of glasses/goggles) can be obtrusive; for example, they can interfere with the field of view, and can be difficult to use when the individual being assessed is wearing their habitual spectacle correction.

### Limitations

We used a treadmill so as to provide a controlled set of walking speeds to determine the effect of changes in phone motion relative to the eyes on the ability to read numbers displayed on a phone. A limitation of using a treadmill is that it eliminates optic flow patterns (Patla 1997) that are present during normal, overground walking. The absence of optic flow information during treadmill walking may influence the ability to read a phone whilst walking and this impact might be different across different walking speeds. Therefore, future research should investigate whether the absence of optic flow in treadmill walking impacts upon phone-reading ability. Another possible limitation of using a treadmill is that the head motion induced by walking on a treadmill may be different to the head motion induced when walking overground for the reasons we highlighted above. However, although there may be differences in the head motion for treadmill walking compared to overground walking, the findings in the current study indicating that PRP is reduced when motion of the phone relative to the eyes increases in irregularity, should be just as applicable to overground walking as to treadmill walking.

### Summary and conclusions

This study has demonstrated that, during walking, PRP is poorer when motion of the phone relative to the eyes becomes increasingly irregular. Irregular relative phone motion arose when walking speed was increased, and when the motion-coupling between the phone and the eyes was further disrupted using an arm brace or when the phone was not hand-held. Our findings suggest that whilst walking, the visual system displays some resistance to motion of the phone relative to the eyes but that considerable inter-individual differences exist in the level of such resistance, even amongst apparently visually normal individuals. As the walking speed was increased or when the motion of the phone relative to the eyes was increased through the use of an arm brace or by not holding the phone in the hand, the visual system was unable to make the necessary eye movements and/or changes in accommodation quickly enough to stabilize and/or focus the retinal image of the irregularly moving phone so as to allow the information presented to be read. We believe these findings may explain why pedestrians slow their walking when viewing their phone. Specifically, walking slows because the head motion induced at slower speeds is smoother and more regular, which means it is easier to couple the motion of the hand-held phone to motion of the head. This coupling ensures that the retinal image of the information on the screen is stable enough to maintain visibility.

## Supplementary Information

Below is the link to the electronic supplementary material.

Supplementary file1 (DOCX 1572 KB)

Supplementary file2 (DOCX 588 KB)

## Data Availability

Phone-reading performance data for every participant is included as Supplementary Material (see Online Resource 2).
