# Supplementary material for: Using a smartphone on the move: do visual constraints explain why we slow walking speed?
Source: Exp Brain Res. 2021 Nov 18;240(2):467–80. doi: 10.1007/s00221-021-06267-6 (PMC8858309; doi:10.1007/s00221-021-06267-6)
Supplement: Supplementary file 1 — Supplementary file1 (DOCX 1572 KB) [file 221_2021_6267_MOESM1_ESM.docx]

**Online Resource 1: Supplementary** **methods**

**How ‘assumed’ gaze angles were determined** **and exemplar data showing calculated gaze angles**

A stationary standing ‘calibration’ trial was recorded for each participant. Each participant stood still on the treadmill and was asked to look to a marker placed at 1.5m in front of them that was vertically and horizontally aligned with the midpoint of their eyes (*Eyes* virtual point). The original head’s reference frame (Fig. S1a) was then relocated to the *Eyes* and rotated to match the participant’s neutral gaze orientation (Fig. S1b), i.e., UD_GAZE_ and RL_GAZE_ angles were configured to be equal to zero when the participant was standing still with their head held in a neutral position and with them looking to the marker in front of them.

With the head’s reference frame embedded at the *Eyes*, the output of the 3D motion of the hand/phone during the reading (dynamic) trials was converted from the lab-based coordinated system into the head’s-reference coordinate system. In other words, the coordinate position (x,y,z) of the phone screen was expressed relative to the *Eyes* position (see Fig. S2 for exemplar data).

The assumed gaze angle in the up-down and in the right-left directions were then determined as follows:

$${UD}_{GAZE}= \tan^{-1} ({D_{z}}/{D_{y}})\cdot({180}/\pi)$$

$${RL}_{GAZE}= \tan^{-1} ({D_{x}}/{D_{y}})\cdot({180}/\pi)$$

where Dx, Dy, and Dz indicate the phone’s relative displacement (in head’s reference frame) in the X (side-to-side), Y (forwards-backwards), and Z (up-down) directions, respectively.

The UD_GAZE_ and RL_GAZE_ angles represent the assumed orientation of gaze during the phone reading task. UD_GAZE_ and RL_GAZE_ angles are positive when the phone is located above or rightwards of where the head is oriented towards, and they become negative when the phone is located below or leftwards of where the head is oriented towards (see Fig. S3 for exemplar data).


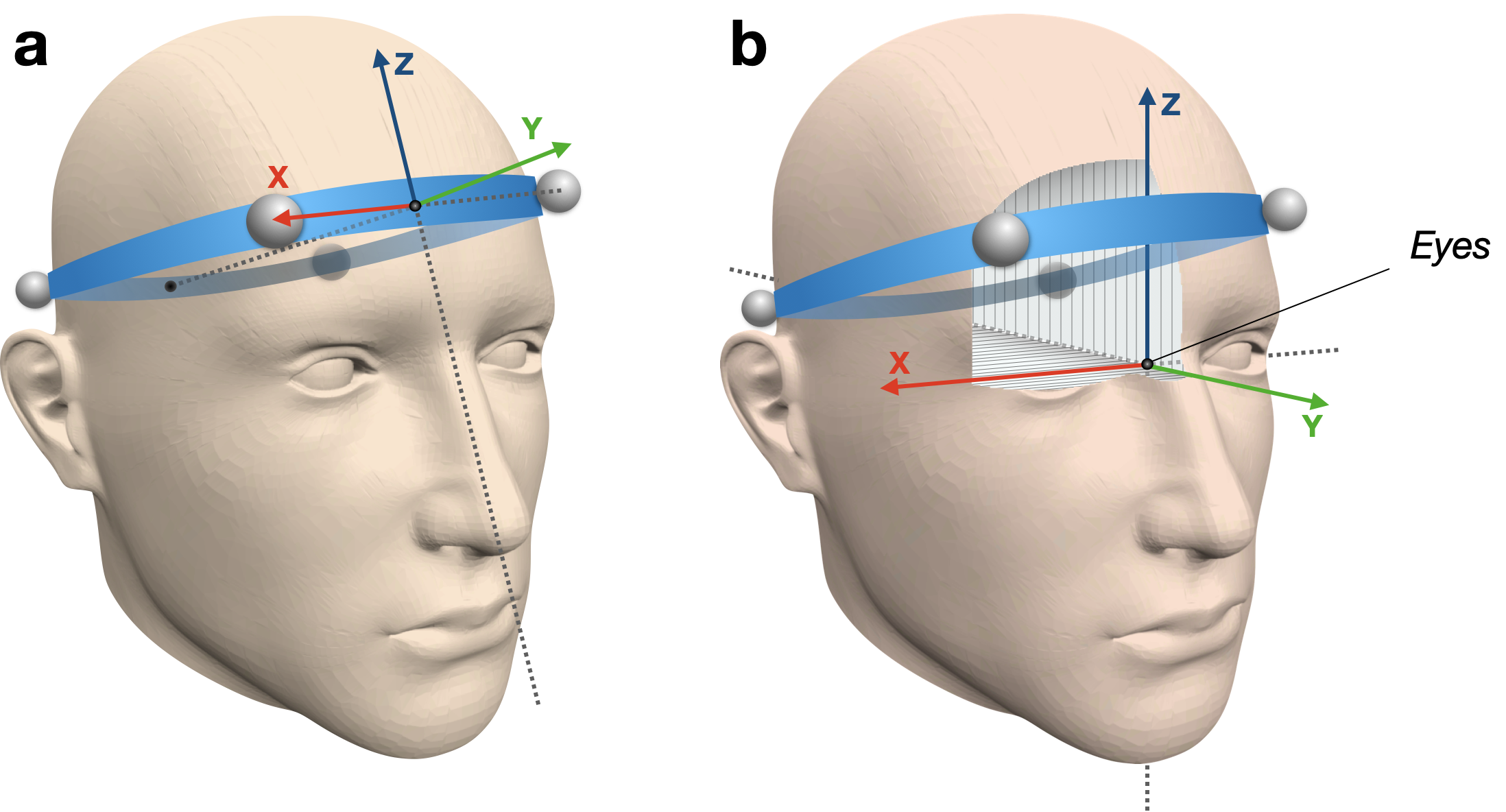


**Fig. S1** Steps taken in relocating and reorienting the PluginGait head-reference frame (**a**) to the *Eyes* virtual point (**b**).


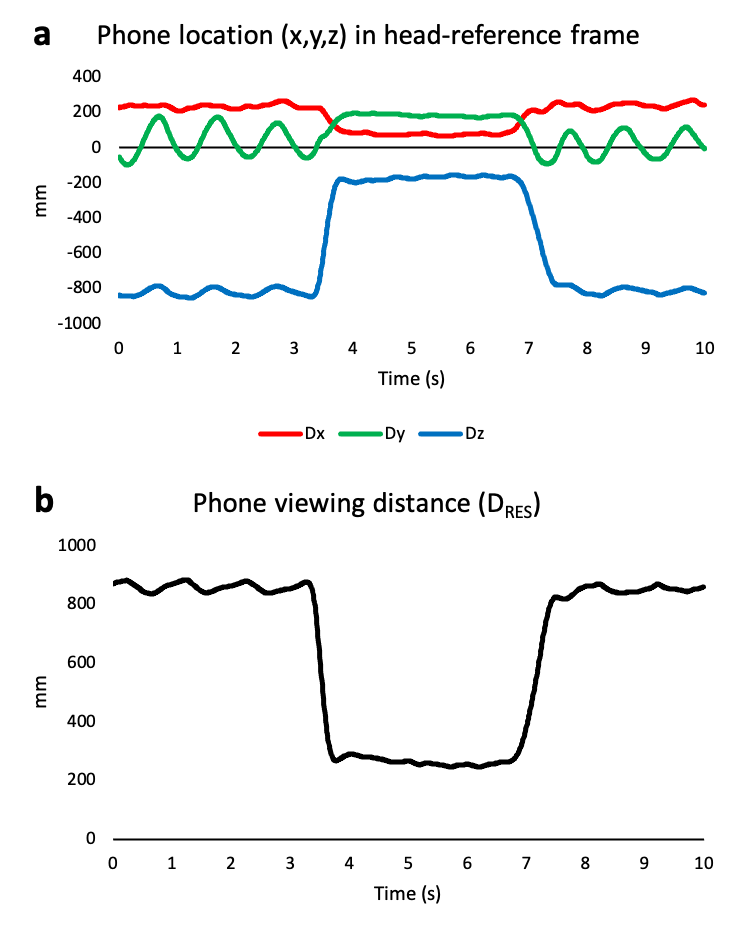


**Fig. S2 a** Exemplar data from one reading trial showing the x, y, z (mm) location of the phone screen in the head-reference frame (i.e., the *Screen* position relative to the *Eyes* position). NB., the negative ‘offset’ in Dz (~800mm) and positive ‘offset’ in Dx (~200mm) before and after the reading period, indicates the hand-held phone is, on average, below and to the right of the *Eyes* position respectively when the arm holding the phone (right arm) swings freely. **b** From the x, y, z location of the phone-screen, the resulting scalar viewing distance (D_RES_) was determined. For this exemplar reading trial the viewing distance was on average around 270 mm.

**
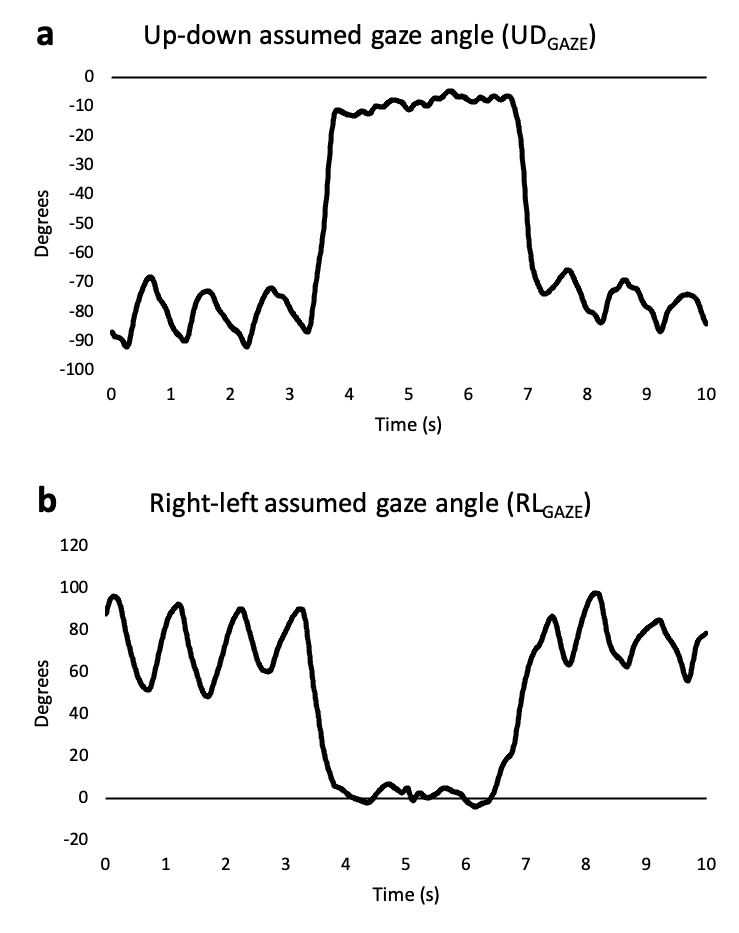
**

**Fig. S3. a)** Illustrates the assumed gaze angle in the up-down direction, and **b)** illustrates the assumed gaze angle in the right-left direction. NB., during the reading period (period from 4-6 sec, for this exemplar trial) the UD_GAZE_ angle approximates to around -10 degrees and the RL_GAZE_ angle approximates to around 5 degrees. This is indicative of the phone being viewed below and slightly to the right of where the head was being oriented towards. The downwards offset is because we naturally view things with a downwards gaze. The slight rightwards ‘offset’ in RL_GAZE_ was because the phone was held in the participant’s right hand, and hence gaze was slightly to the right of where the head was being oriented towards.
