# Supplementary material for: Using a smartphone on the move: do visual constraints explain why we slow walking speed?
Source: Exp Brain Res. 2021 Nov 18;240(2):467–80. doi: 10.1007/s00221-021-06267-6 (PMC8858309; doi:10.1007/s00221-021-06267-6)
Supplement: Supplementary file 2 — Supplementary file2 (DOCX 588 KB) [file 221_2021_6267_MOESM2_ESM.docx]

**Supplementary Material**

**Methods**:

**How assumed gaze angles were determined**

A stationary standing ‘calibration’ trial was recorded for each participant. Each participant stood still on the treadmill and was asked to look to a marker placed at 1.5m in front of them that was vertically and horizontally aligned with the midpoint of their eyes (*Eyes* virtual point). The head’s reference frame was then relocated to the *Eyes* and then rotated to match the participant’s neutral gaze orientation (Figure S1), i.e., UD_GAZE_ and RL_GAZE_ angles were configured to be equal to zero when the participant was standing still with their head held in a neutral position and looking to the marker in front of them.

With the head’s reference frame embedded at the *Eyes*, the output of the 3D motion tracking from the reading (dynamic) trials was converted from the lab-based coordinated system into the head’s-reference coordinate system. In other words, the coordinate position (x,y,z) of the phone screen was determined relative to the *Eyes* position (see Figure S2 for exemplar data).

The assumed gaze angle in the up-down and in the right-left directions were then determined as follos:

${UD}_{GAZE}= \tan^{-1} ({D_{z}}/{D_{y}})\cdot({180}/\pi)$

${RL}_{GAZE}= \tan^{-1} ({D_{x}}/{D_{y}})\cdot({180}/\pi)$.

where Dx, Dy, and Dz indicate the relative phone displacement (in head’s reference frame) in the X, Y, and Z directions, respectively.

The UD_GAZE_ and RL_GAZE_ angles represent the assumed orientation of gaze during the phone reading task. UD_GAZE_ and RL_GAZE_ angles are positive when the phone is located above or rightwards of a neutral head orientation, and they become negative when the phone is located below or leftwards of the neutral head orientation (see Figure S3 for exemplar data).


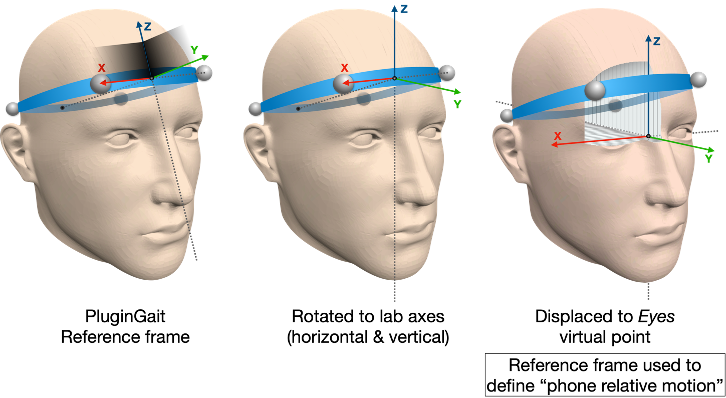


**Fig. S1** Steps taken in relocating and reorienting the PluginGait head-reference frame to the *Eyes* virtual point.


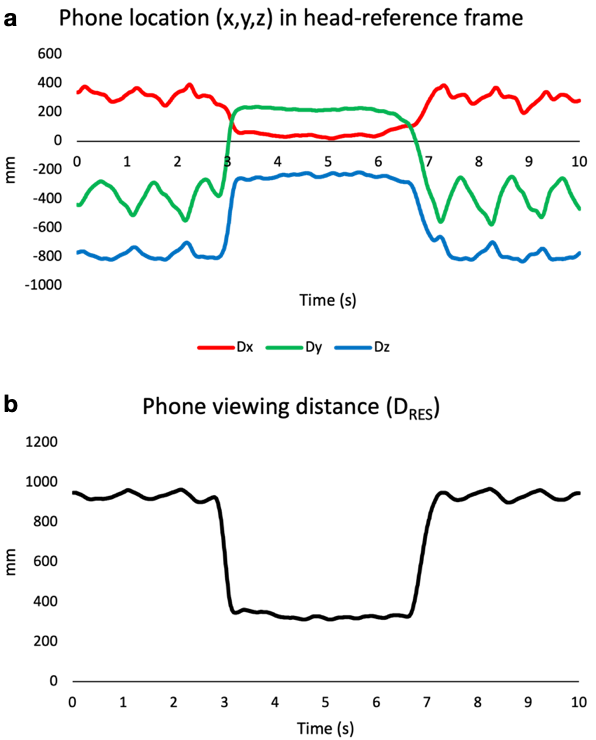


**Fig. S2 a** Exemplar data from one reading trial showing the x, y, z (mm) location of the phone screen in the head-reference frame (i.e., the *Screen* position relative to the *Eyes* position). NB., the negative ‘offset’ in Dy (~400mm) and Dz (~800mm) and positive ‘offset’ in Dx (~30mm) before and after the reading period, indicates the hand-held phone is, on average, behind, below and to the right of the *Eyes* position respectively when the arm holding the phone (right arm) swings freely. **b** From the x, y, z location of the phone-screen, the resulting scalar viewing distance (D_RES_) was determined. For this exemplar reading trial the viewing distance was on average around 320mm.

**
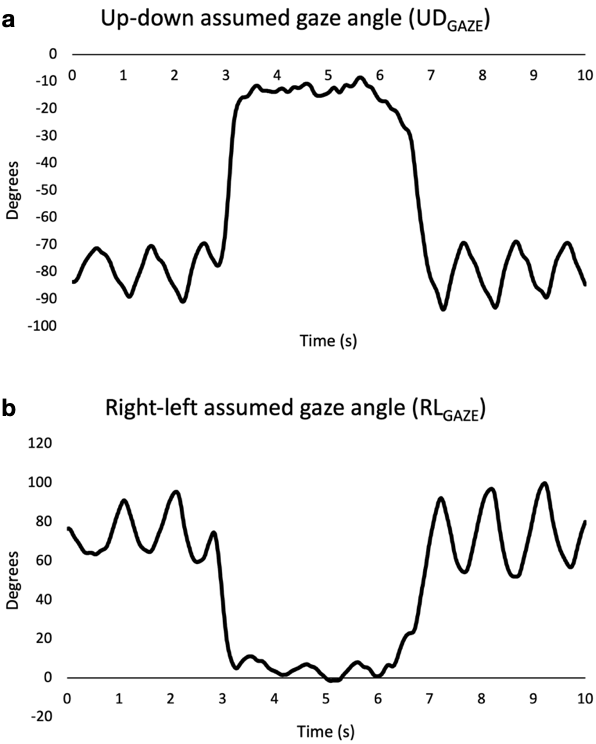
**

**Fig. S3 a** illustrates the assumed gaze angle in the up-down direction, and **b** illustrates the assumed gaze angle in the right-left direction. NB., during the reading period (period from 3.5-5.5 sec, for this exemplar trial) the UD_GAZE_ angle approximates to around -15 degrees and the RL_GAZE_ angle approximates to around 5 degrees. This is indicative of the head being orientated forwards and tilted slightly downwards with the phone being viewed slightly to the right of the head. The slight rightwards ‘offset’ in RL_GAZE_ was because the phone was held in the participant’s right hand. The downwards ‘offset’ in UD_GAZE_ indicates the phone was being held at an average height that was below the neutral gaze orientation.

**Results**: auxiliary

**
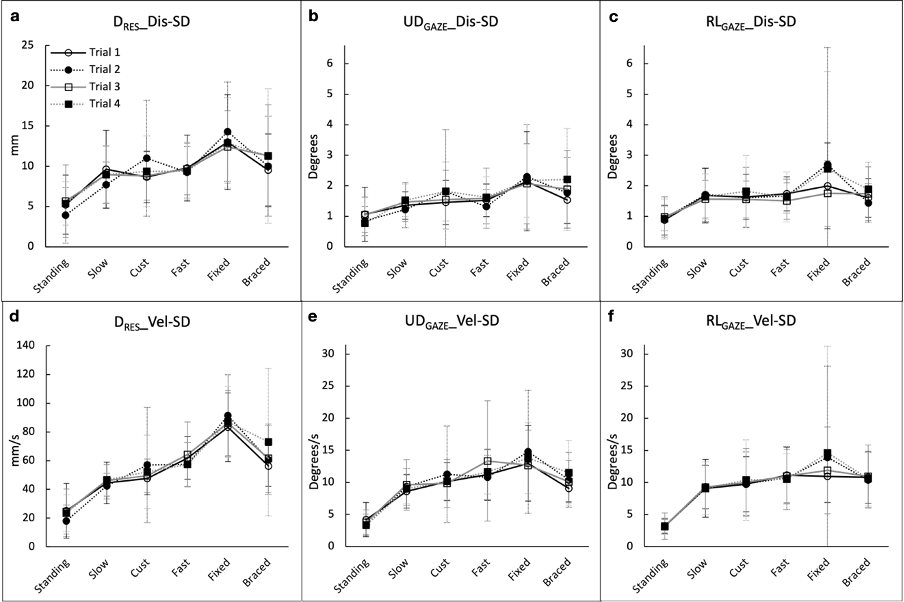
**

**Fig. 4** Group average variability in the phone’s relative displacement **a** D_RES__Dis-SD, **b** UD_GAZE__Dis-SD, **c** RL_GAZE__Dis-SD, and group average variability in the phone’s relative velocity; **d** D_RES__Vel-SD, **e** UD_GAZE__Vel-SD, **f** RL_GAZE__Vel-SD, across testing conditions and trial repetitions. Error bars represent the group SD for each condition and trial.

**Table 2** Database containing each participant’s phone reading performance scores (see column PRP) for every condition and trial. PRP represents the number of phone-digits correctly read (out of 11) during the 2-second reading period.

| **P** | **C** | **T** | **PRP** |
| --- | --- | --- | --- |
| 1 | S0 | 1 | 11 |
| 1 | S0 | 2 | 11 |
| 1 | S0 | 3 | 11 |
| 1 | S0 | 4 | 11 |
| 1 | HS | 1 | 8 |
| 1 | HS | 2 | 11 |
| 1 | HS | 3 | 11 |
| 1 | HS | 4 | 11 |
| 1 | HN | 1 | 8 |
| 1 | HN | 2 | 11 |
| 1 | HN | 3 | 11 |
| 1 | HN | 4 | 11 |
| 1 | HF | 1 | 10 |
| 1 | HF | 2 | 11 |
| 1 | HF | 3 | 11 |
| 1 | HF | 4 | 11 |
| 1 | SF | 1 | 11 |
| 1 | SF | 2 | 11 |
| 1 | SF | 3 | 11 |
| 1 | SF | 4 | 11 |
| 1 | BF | 1 | 5 |
| 1 | BF | 2 | 8 |
| 1 | BF | 3 | 9 |
| 1 | BF | 4 | 11 |
| 2 | S0 | 1 | 5 |
| 2 | S0 | 2 | 6 |
| 2 | S0 | 3 | 6 |
| 2 | S0 | 4 | 8 |
| 2 | HS | 1 | 11 |
| 2 | HS | 2 | 11 |
| 2 | HS | 3 | 9 |
| 2 | HS | 4 | 5 |
| 2 | HN | 1 | 7 |
| 2 | HN | 2 | 7 |
| 2 | HN | 3 | 8 |
| 2 | HN | 4 | 7 |
| 2 | HF | 1 | 11 |
| 2 | HF | 2 | 5 |
| 2 | HF | 3 | 9 |
| 2 | HF | 4 | 7 |
| 2 | SF | 1 | 6 |
| 2 | SF | 2 | 9 |
| 2 | SF | 3 | 7 |
| 2 | SF | 4 | 8 |
| 2 | BF | 1 | 8 |
| 2 | BF | 2 | 11 |
| 2 | BF | 3 | 9 |
| 2 | BF | 4 | 8 |
| 3 | S0 | 1 | 9 |
| 3 | S0 | 2 | 9 |
| 3 | S0 | 3 | 9 |
| 3 | S0 | 4 | 10 |
| 3 | HS | 1 | 9 |
| 3 | HS | 2 | 8 |
| 3 | HS | 3 | 9 |
| 3 | HS | 4 | 9 |
| 3 | HN | 1 | 9 |
| 3 | HN | 2 | 9 |
| 3 | HN | 3 | 8 |
| 3 | HN | 4 | 9 |
| 3 | HF | 1 | 8 |
| 3 | HF | 2 | 9 |
| 3 | HF | 3 | 9 |
| 3 | HF | 4 | 9 |
| 3 | SF | 1 | 8 |
| 3 | SF | 2 | 8 |
| 3 | SF | 3 | 9 |
| 3 | SF | 4 | 5 |
| 3 | BF | 1 | 3 |
| **P** | **C** | **T** | **PRP** |
| 3 | BF | 2 | 9 |
| 3 | BF | 3 | 6 |
| 3 | BF | 4 | 7 |
| 4 | S0 | 1 | 8 |
| 4 | S0 | 2 | 11 |
| 4 | S0 | 3 | 5 |
| 4 | S0 | 4 | 11 |
| 4 | HS | 1 | 8 |
| 4 | HS | 2 | 11 |
| 4 | HS | 3 | 11 |
| 4 | HS | 4 | 11 |
| 4 | HN | 1 | 11 |
| 4 | HN | 2 | 11 |
| 4 | HN | 3 | 11 |
| 4 | HN | 4 | 11 |
| 4 | HF | 1 | 11 |
| 4 | HF | 2 | 11 |
| 4 | HF | 3 | 11 |
| 4 | HF | 4 | 11 |
| 4 | SF | 1 | 11 |
| 4 | SF | 2 | 11 |
| 4 | SF | 3 | 11 |
| 4 | SF | 4 | 8 |
| 4 | BF | 1 | 11 |
| 4 | BF | 2 | 5 |
| 4 | BF | 3 | 11 |
| 4 | BF | 4 | 11 |
| 5 | S0 | 1 | 11 |
| 5 | S0 | 2 | 9 |
| 5 | S0 | 3 | 11 |
| 5 | S0 | 4 | 8 |
| 5 | HS | 1 | 9 |
| 5 | HS | 2 | 11 |
| 5 | HS | 3 | 11 |
| 5 | HS | 4 | 11 |
| 5 | HN | 1 | 8 |
| 5 | HN | 2 | 11 |
| 5 | HN | 3 | 8 |
| 5 | HN | 4 | 11 |
| 5 | HF | 1 | 11 |
| 5 | HF | 2 | 9 |
| 5 | HF | 3 | 11 |
| 5 | HF | 4 | 11 |
| 5 | SF | 1 | 8 |
| 5 | SF | 2 | 8 |
| 5 | SF | 3 | 11 |
| 5 | SF | 4 | 8 |
| 5 | BF | 1 | 7 |
| 5 | BF | 2 | 8 |
| 5 | BF | 3 | 11 |
| 5 | BF | 4 | 11 |
| 6 | S0 | 1 | 11 |
| 6 | S0 | 2 | 11 |
| 6 | S0 | 3 | 11 |
| 6 | S0 | 4 | 9 |
| 6 | HS | 1 | 11 |
| 6 | HS | 2 | 11 |
| 6 | HS | 3 | 11 |
| 6 | HS | 4 | 9 |
| 6 | HN | 1 | 10 |
| 6 | HN | 2 | 11 |
| 6 | HN | 3 | 11 |
| 6 | HN | 4 | 11 |
| 6 | HF | 1 | 7 |
| 6 | HF | 2 | 8 |
| 6 | HF | 3 | 8 |
| 6 | HF | 4 | 8 |
| 6 | SF | 1 | 5 |
| 6 | SF | 2 | 8 |
| **P** | **C** | **T** | **PRP** |
| 6 | SF | 3 | 9 |
| 6 | SF | 4 | 5 |
| 6 | BF | 1 | 5 |
| 6 | BF | 2 | 7 |
| 6 | BF | 3 | 5 |
| 6 | BF | 4 | 6 |
| 7 | S0 | 1 | 7 |
| 7 | S0 | 2 | 9 |
| 7 | S0 | 3 | 11 |
| 7 | S0 | 4 | 9 |
| 7 | HS | 1 | 8 |
| 7 | HS | 2 | 11 |
| 7 | HS | 3 | 11 |
| 7 | HS | 4 | 11 |
| 7 | HN | 1 | 7 |
| 7 | HN | 2 | 7 |
| 7 | HN | 3 | 9 |
| 7 | HN | 4 | 11 |
| 7 | HF | 1 | 7 |
| 7 | HF | 2 | 5 |
| 7 | HF | 3 | 11 |
| 7 | HF | 4 | 10 |
| 7 | SF | 1 | 7 |
| 7 | SF | 2 | 7 |
| 7 | SF | 3 | 5 |
| 7 | SF | 4 | 2 |
| 7 | BF | 1 | 5 |
| 7 | BF | 2 | 9 |
| 7 | BF | 3 | 9 |
| 7 | BF | 4 | 2 |
| 8 | S0 | 1 | 6 |
| 8 | S0 | 2 | 8 |
| 8 | S0 | 3 | 7 |
| 8 | S0 | 4 | 7 |
| 8 | HS | 1 | 3 |
| 8 | HS | 2 | 3 |
| 8 | HS | 3 | 6 |
| 8 | HS | 4 | 5 |
| 8 | HN | 1 | 5 |
| 8 | HN | 2 | 5 |
| 8 | HN | 3 | 5 |
| 8 | HN | 4 | 5 |
| 8 | HF | 1 | 5 |
| 8 | HF | 2 | 8 |
| 8 | HF | 3 | 5 |
| 8 | HF | 4 | 5 |
| 8 | SF | 1 | 5 |
| 8 | SF | 2 | 5 |
| 8 | SF | 3 | 5 |
| 8 | SF | 4 | 7 |
| 8 | BF | 1 | 5 |
| 8 | BF | 2 | 5 |
| 8 | BF | 3 | 4 |
| 8 | BF | 4 | 5 |
| 9 | S0 | 1 | 11 |
| 9 | S0 | 2 | 11 |
| 9 | S0 | 3 | 11 |
| 9 | S0 | 4 | 11 |
| 9 | HS | 1 | 11 |
| 9 | HS | 2 | 11 |
| 9 | HS | 3 | 5 |
| 9 | HS | 4 | 7 |
| 9 | HN | 1 | 11 |
| 9 | HN | 2 | 11 |
| 9 | HN | 3 | 9 |
| 9 | HN | 4 | 8 |
| 9 | HF | 1 | 8 |
| 9 | HF | 2 | 11 |
| 9 | HF | 3 | 11 |
| **P** | **C** | **T** | **PRP** |
| 9 | HF | 4 | 11 |
| 9 | SF | 1 | 9 |
| 9 | SF | 2 | 9 |
| 9 | SF | 3 | 11 |
| 9 | SF | 4 | 6 |
| 9 | BF | 1 | 8 |
| 9 | BF | 2 | 5 |
| 9 | BF | 3 | 8 |
| 9 | BF | 4 | 11 |
| 10 | S0 | 1 | 8 |
| 10 | S0 | 2 | 8 |
| 10 | S0 | 3 | 8 |
| 10 | S0 | 4 | 7 |
| 10 | HS | 1 | 8 |
| 10 | HS | 2 | 6 |
| 10 | HS | 3 | 9 |
| 10 | HS | 4 | 8 |
| 10 | HN | 1 | 5 |
| 10 | HN | 2 | 5 |
| 10 | HN | 3 | 5 |
| 10 | HN | 4 | 7 |
| 10 | HF | 1 | 5 |
| 10 | HF | 2 | 4 |
| 10 | HF | 3 | 6 |
| 10 | HF | 4 | 9 |
| 10 | SF | 1 | 8 |
| 10 | SF | 2 | 8 |
| 10 | SF | 3 | 5 |
| 10 | SF | 4 | 9 |
| 10 | BF | 1 | 5 |
| 10 | BF | 2 | 5 |
| 10 | BF | 3 | 7 |
| 10 | BF | 4 | 5 |
| 11 | S0 | 1 | 11 |
| 11 | S0 | 2 | 11 |
| 11 | S0 | 3 | 11 |
| 11 | S0 | 4 | 11 |
| 11 | HS | 1 | 11 |
| 11 | HS | 2 | 8 |
| 11 | HS | 3 | 11 |
| 11 | HS | 4 | 11 |
| 11 | HN | 1 | 9 |
| 11 | HN | 2 | 11 |
| 11 | HN | 3 | 9 |
| 11 | HN | 4 | 7 |
| 11 | HF | 1 | 11 |
| 11 | HF | 2 | 6 |
| 11 | HF | 3 | 11 |
| 11 | HF | 4 | 11 |
| 11 | SF | 1 | 11 |
| 11 | SF | 2 | 7 |
| 11 | SF | 3 | 9 |
| 11 | SF | 4 | 11 |
| 11 | BF | 1 | 5 |
| 11 | BF | 2 | 7 |
| 11 | BF | 3 | 7 |
| 11 | BF | 4 | 11 |
| 12 | S0 | 1 | 8 |
| 12 | S0 | 2 | 11 |
| 12 | S0 | 3 | 8 |
| 12 | S0 | 4 | 11 |
| 12 | HS | 1 | 9 |
| 12 | HS | 2 | 11 |
| 12 | HS | 3 | 9 |
| 12 | HS | 4 | 5 |
| 12 | HN | 1 | 8 |
| 12 | HN | 2 | 9 |
| 12 | HN | 3 | 11 |
| 12 | HN | 4 | 8 |
| **P** | **C** | **T** | **PRP** |
| 12 | HF | 1 | 8 |
| 12 | HF | 2 | 7 |
| 12 | HF | 3 | 7 |
| 12 | HF | 4 | 9 |
| 12 | SF | 1 | 5 |
| 12 | SF | 2 | 8 |
| 12 | SF | 3 | 11 |
| 12 | SF | 4 | 11 |
| 12 | BF | 1 | 8 |
| 12 | BF | 2 | 10 |
| 12 | BF | 3 | 7 |
| 12 | BF | 4 | 7 |
| 13 | S0 | 1 | 3 |
| 13 | S0 | 2 | 3 |
| 13 | S0 | 3 | 3 |
| 13 | S0 | 4 | 3 |
| 13 | HS | 1 | 3 |
| 13 | HS | 2 | 3 |
| 13 | HS | 3 | 3 |
| 13 | HS | 4 | 3 |
| 13 | HN | 1 | 3 |
| 13 | HN | 2 | 4 |
| 13 | HN | 3 | 5 |
| 13 | HN | 4 | 5 |
| 13 | HF | 1 | 6 |
| 13 | HF | 2 | 8 |
| 13 | HF | 3 | 7 |
| 13 | HF | 4 | 5 |
| 13 | SF | 1 | 3 |
| 13 | SF | 2 | 4 |
| 13 | SF | 3 | 4 |
| 13 | SF | 4 | 4 |
| 13 | BF | 1 | 7 |
| 13 | BF | 2 | 8 |
| 13 | BF | 3 | 8 |
| 13 | BF | 4 | 9 |
| 14 | S0 | 1 | 11 |
| 14 | S0 | 2 | 9 |
| 14 | S0 | 3 | 9 |
| 14 | S0 | 4 | 8 |
| 14 | HS | 1 | 11 |
| 14 | HS | 2 | 5 |
| 14 | HS | 3 | 11 |
| 14 | HS | 4 | 9 |
| 14 | HN | 1 | 11 |
| 14 | HN | 2 | 11 |
| 14 | HN | 3 | 11 |
| 14 | HN | 4 | 9 |
| 14 | HF | 1 | 7 |
| 14 | HF | 2 | 7 |
| 14 | HF | 3 | 7 |
| 14 | HF | 4 | 8 |
| 14 | SF | 1 | 8 |
| 14 | SF | 2 | 11 |
| 14 | SF | 3 | 8 |
| 14 | SF | 4 | 9 |
| 14 | BF | 1 | 5 |
| 14 | BF | 2 | 5 |
| 14 | BF | 3 | 7 |
| 14 | BF | 4 | 7 |
| 15 | S0 | 1 | 11 |
| 15 | S0 | 2 | 11 |
| 15 | S0 | 3 | 9 |
| 15 | S0 | 4 | 9 |
| 15 | HS | 1 | 8 |
| 15 | HS | 2 | 9 |
| 15 | HS | 3 | 8 |
| 15 | HS | 4 | 10 |
| 15 | HN | 1 | 8 |
| **P** | **C** | **T** | **PRP** |
| 15 | HN | 2 | 11 |
| 15 | HN | 3 | 9 |
| 15 | HN | 4 | 11 |
| 15 | HF | 1 | 11 |
| 15 | HF | 2 | 7 |
| 15 | HF | 3 | 11 |
| 15 | HF | 4 | 10 |
| 15 | SF | 1 | 8 |
| 15 | SF | 2 | 8 |
| 15 | SF | 3 | 11 |
| 15 | SF | 4 | 7 |
| 15 | BF | 1 | 8 |
| 15 | BF | 2 | 9 |
| 15 | BF | 3 | 9 |
| 15 | BF | 4 | 8 |
| 16 | S0 | 1 | 8 |
| 16 | S0 | 2 | 9 |
| 16 | S0 | 3 | 8 |
| 16 | S0 | 4 | 9 |
| 16 | HS | 1 | 9 |
| 16 | HS | 2 | 11 |
| 16 | HS | 3 | 11 |
| 16 | HS | 4 | 11 |
| 16 | HN | 1 | 11 |
| 16 | HN | 2 | 11 |
| 16 | HN | 3 | 5 |
| 16 | HN | 4 | 11 |
| 16 | HF | 1 | 9 |
| 16 | HF | 2 | 11 |
| 16 | HF | 3 | 9 |
| 16 | HF | 4 | 7 |
| 16 | SF | 1 | 9 |
| 16 | SF | 2 | 11 |
| 16 | SF | 3 | 11 |
| 16 | SF | 4 | 11 |
| 16 | BF | 1 | 7 |
| 16 | BF | 2 | 7 |
| 16 | BF | 3 | 10 |
| 16 | BF | 4 | 11 |
| 17 | S0 | 1 | 11 |
| 17 | S0 | 2 | 11 |
| 17 | S0 | 3 | 11 |
| 17 | S0 | 4 | 11 |
| 17 | HS | 1 | 11 |
| 17 | HS | 2 | 11 |
| 17 | HS | 3 | 11 |
| 17 | HS | 4 | 11 |
| 17 | HN | 1 | 11 |
| 17 | HN | 2 | 9 |
| 17 | HN | 3 | 11 |
| 17 | HN | 4 | 11 |
| 17 | HF | 1 | 8 |
| 17 | HF | 2 | 9 |
| 17 | HF | 3 | 11 |
| 17 | HF | 4 | 11 |
| 17 | SF | 1 | 11 |
| 17 | SF | 2 | 11 |
| 17 | SF | 3 | 9 |
| 17 | SF | 4 | 11 |
| 17 | BF | 1 | 11 |
| 17 | BF | 2 | 11 |
| 17 | BF | 3 | 8 |
| 17 | BF | 4 | 11 |
| 18 | S0 | 1 | 9 |
| 18 | S0 | 2 | 11 |
| 18 | S0 | 3 | 10 |
| 18 | S0 | 4 | 10 |
| 18 | HS | 1 | 11 |
| 18 | HS | 2 | 11 |
| **P** | **C** | **T** | **PRP** |
| 18 | HS | 3 | 10 |
| 18 | HS | 4 | 11 |
| 18 | HN | 1 | 11 |
| 18 | HN | 2 | 11 |
| 18 | HN | 3 | 11 |
| 18 | HN | 4 | 10 |
| 18 | HF | 1 | 9 |
| 18 | HF | 2 | 10 |
| 18 | HF | 3 | 11 |
| 18 | HF | 4 | 11 |
| 18 | SF | 1 | 11 |
| 18 | SF | 2 | 9 |
| 18 | SF | 3 | 11 |
| 18 | SF | 4 | 11 |
| 18 | BF | 1 | 8 |
| 18 | BF | 2 | 11 |
| 18 | BF | 3 | 9 |
| 18 | BF | 4 | 11 |
| 19 | S0 | 1 | 11 |
| 19 | S0 | 2 | 9 |
| 19 | S0 | 3 | 11 |
| 19 | S0 | 4 | 11 |
| 19 | HS | 1 | 11 |
| 19 | HS | 2 | 11 |
| 19 | HS | 3 | 11 |
| 19 | HS | 4 | 11 |
| 19 | HN | 1 | 11 |
| 19 | HN | 2 | 9 |
| 19 | HN | 3 | 11 |
| 19 | HN | 4 | 9 |
| 19 | HF | 1 | 11 |
| 19 | HF | 2 | 11 |
| 19 | HF | 3 | 11 |
| 19 | HF | 4 | 9 |
| 19 | SF | 1 | 8 |
| 19 | SF | 2 | 11 |
| 19 | SF | 3 | 11 |
| 19 | SF | 4 | 11 |
| 19 | BF | 1 | 11 |
| 19 | BF | 2 | 10 |
| 19 | BF | 3 | 11 |
| 19 | BF | 4 | 11 |
| 20 | S0 | 1 | 8 |
| 20 | S0 | 2 | 9 |
| 20 | S0 | 3 | 8 |
| 20 | S0 | 4 | 8 |
| 20 | HS | 1 | 11 |
| 20 | HS | 2 | 8 |
| 20 | HS | 3 | 9 |
| 20 | HS | 4 | 8 |
| 20 | HN | 1 | 9 |
| 20 | HN | 2 | 9 |
| 20 | HN | 3 | 7 |
| 20 | HN | 4 | 8 |
| 20 | HF | 1 | 7 |
| 20 | HF | 2 | 7 |
| 20 | HF | 3 | 5 |
| 20 | HF | 4 | 8 |
| 20 | SF | 1 | 7 |
| 20 | SF | 2 | 9 |
| 20 | SF | 3 | 8 |
| 20 | SF | 4 | 8 |
| 20 | BF | 1 | 7 |
| 20 | BF | 2 | 7 |
| 20 | BF | 3 | 8 |
| 20 | BF | 4 | 9 |

“P”: Participant, “C”: Condition, “T”: Trial, “PRP”: Phone Reading Performance, “S0”: Standing, “HS”: Slow, “HN”: Normal, “HF”: Fast, “SF”: Fixed, “BF”: Braced.
